# Peritoneal Carcinomatosis as an Initial Presentation of Gastrointestinal Stromal Tumor: A Case Report and Review of the Literature

**DOI:** 10.7759/cureus.26421

**Published:** 2022-06-29

**Authors:** Gonca Ozcan, Garima Gautam, Teresa Da Cunha, Erica C Becker, Nikola Perosevic

**Affiliations:** 1 Department of Internal Medicine, University of Connecticut Health, Farmington, USA; 2 Department of Internal Medicine, University of Connecticut Health, Hartford, USA; 3 Internal Medicine, Saint Francis Hospital, Hartford, USA

**Keywords:** metastatic gastrointestinal stromal tumor, tyrosine kinase inhibitors (tki), gastrointestinal malignancy, peritoneal carcinomatosis, gastrointestinal stromal tumor (gist)

## Abstract

Gastrointestinal stromal tumors (GISTs) are a rare type of tumor with a high risk of malignant transformation. The majority of GISTs are asymptomatic. Surgical resection remains the mainstay of treatment given that GIST is resistant to traditional chemotherapy and radiotherapy. In the last two decades, the discovery of targeted therapy with tyrosine kinase inhibitor therapy (TKI) and widespread mutation analysis of tumors have transformed the treatment of GIST. We present a case of a patient in whom imaging findings were consistent with carcinomatous peritonitis concerning a gynecological malignancy but who was later found to have an unresectable GIST which locally regressed with TKI.

## Introduction

Gastrointestinal stromal tumors (GISTs) are non-epithelial stromal tumors with an incidence of 1-1.5 per 100,000 per year and a prevalence of 13 per 100,000, despite being the most common gastrointestinal mesenchymal tumors in adults [1]. GIST can present with gastrointestinal bleeding or mass-related symptoms but is most commonly clinically silent, making the diagnosis challenging. The median age of diagnosis is 60 years old, and there is no significant sex predominance [1,2]. More than 90% of GISTs have an activating receptor tyrosine kinase (KIT) or platelet-derived growth factor receptor alpha (PDGFRA) mutation [3]. Surgery remains the mainstay of treatment for resectable tumors. Targeted therapy with tyrosine kinase inhibitors (TKI) can lead to a dramatic response in unresectable tumors. Our case was presented as a poster at the Society of General Internal Medicine meeting in 2020.

## Case presentation

A 74-year-old female with a history of adrenal insufficiency, diabetes mellitus, and hypothyroidism presented with severe nausea, vomiting, and abdominal pain for a week. On admission, her vital signs were within normal range. The physical exam was only remarkable for generalized abdominal tenderness without rebound or guarding. The laboratory workup was only remarkable for mild hyponatremia (Na: 132 mmol/L) but otherwise normal.

**Figure 1:**
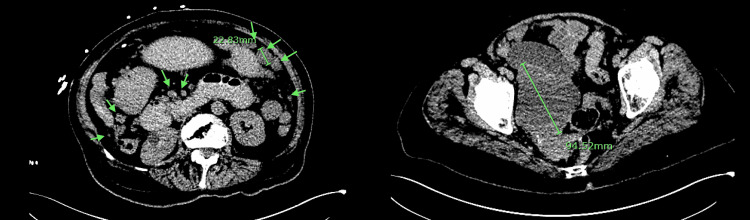
Computed tomography of abdomen pelvis without IV contrast at the time of diagnoses showing multiple masses throughout the abdomen and pelvis, consistent with peritoneal studding and possible primary mass in the right pelvic region which measures 9.4 cm x 7.1 cm

Due to concerns of small bowel obstruction, a computed tomography (CT) of the abdomen was obtained and showed high-grade small bowel obstruction with possible transition in the right pelvis. There were multiple masses throughout the abdomen and pelvis, consistent with peritoneal studding and a possible primary mass in the right pelvic region that was concerned to be ovarian in origin (Figure 1).

**Figure 2:**
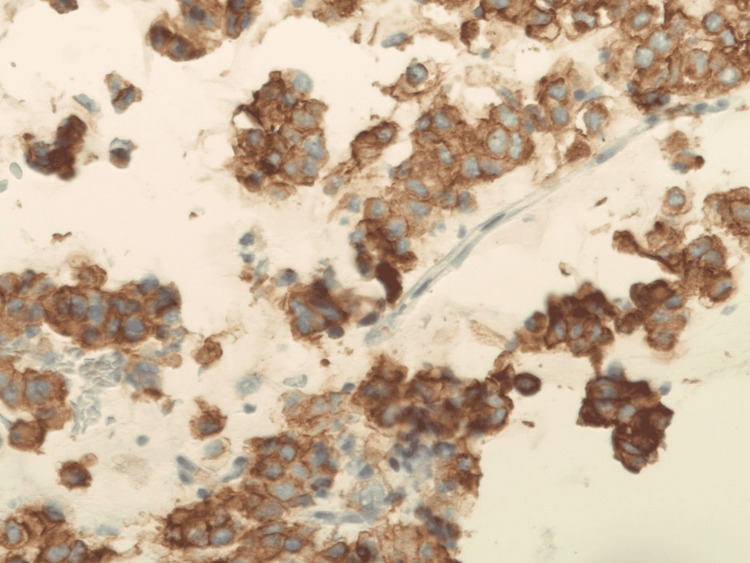
Immunohistochemical staining showing strongly CD117 positive tumor

**Figure 3:**
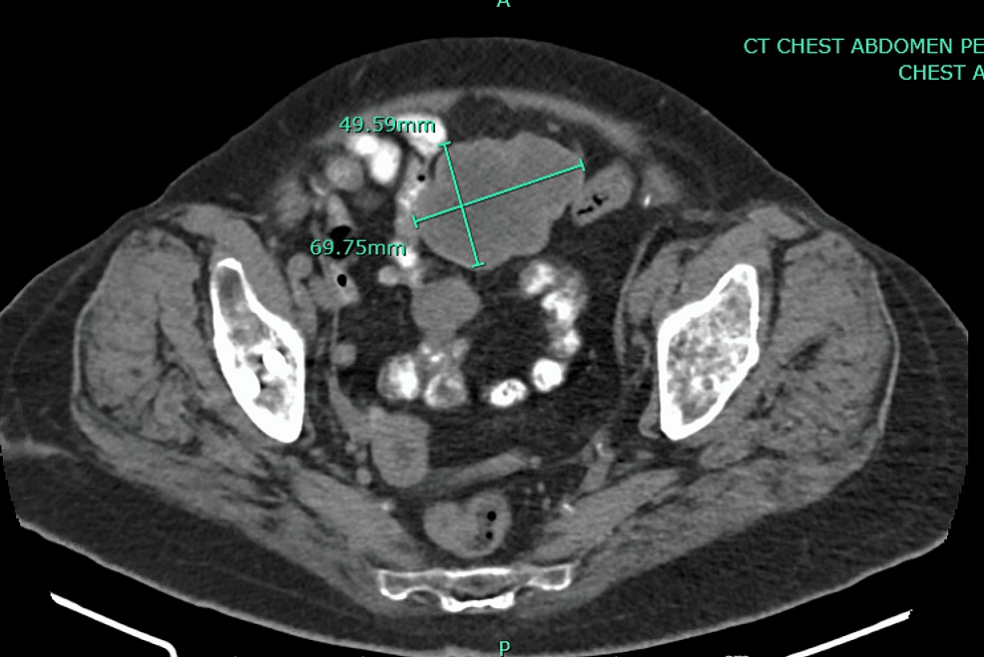
Computed tomography without IV contrast showing regression of primary mass measuring 4.9 cm × 6.9 cm

A nasogastric tube was placed at the bedside for decompression. ​​Tumor markers revealed a CA-125 antigen that was slightly elevated to 67.2 U/ml (range 0-35 U/ml); CA-19-9 and CEA antigens were normal. The patient has not been deemed a candidate for surgical intervention, given the extensive disease upon surgical evaluation. The patient subsequently underwent a CT-guided omental biopsy, which showed the tumor cells were epithelioid and contained abundant clear cytoplasm with vesicular chromatin and vacuoles in the nuclei. Immunohistochemical studies were positive for cKit, DOG1, CD34, vimentin, and focally positive for EMA (Figure 2). However, they were negative for pan-keratin, CK7, CK20, desmin, Pax8, S100, WT-1, and PLAP. This was consistent with the diagnosis of GI stromal tumor. The primary lesion is thought to be the largest pelvic mass (Figures 1-3).

The patient was initiated on palliative therapy with Imatinib 400 mg daily. A subsequent CT scan of the abdomen showed several discrete nodules and masses along with bowel loops consistent with the patient's known GIST tumor. The largest lesion is in the pelvis and measures 5.3 cm × 8.7 cm in axial size. She tolerated the treatment well, and her repeat CT scan of the abdomen and pelvis at nine months showed overall regression of stromal tumor nodules and primary lesion (Figure 3). The patient remained on active therapy with Imatinib 400 mg daily for 15 months and tolerated therapy well. After 15 months, the patient preferred to stop taking Imatinib as she opted for naturopathic treatment and lost follow-up.

## Discussion

GIST mostly originates from the interstitial cells of Cajal (ICC), the gastrointestinal tract's pacemaker cells that control motility [4,5]. The predominant localization of GIST is the stomach (60%), followed by the small intestine (30-20%). Similar to our patient's developing GIST in the omentum, GISTs may develop in the colorectum, esophagus, and rarely, in the mesentery or retroperitoneum (extragastrointestinal GIST) [1,5]. In many cases, GISTs are an accidental finding; however, they can present with upper gastrointestinal bleeding and abdominal discomfort or symptoms similar to PUD [1]. Our patient presented with nonspecific symptoms of nausea and vomiting, and due to the imaging findings, the initial concern was ovarian malignancy with widespread metastasis intra-abdominal carcinomatosis that was causing paralytic ileus and/or small bowel obstruction. There is no proven screening method for GIST given the rarity of diagnosis and cost-effectiveness concerns [4].

Imaging findings of GISTs vary depending on the size and stage of the tumor and time of presentation. Primary GISTs are typically large, hypervascular, enhancing masses on contrast-enhanced CT scans and are often heterogeneous due to necrosis, hemorrhage, or cystic degeneration at the time of presentation [6]. Response to treatment or monitoring disease for recurrence can be assessed with CT or positron emission tomography [7].

Overall, 95% of "classic" GISTs overexpress CD117 (KIT) by immunohistochemistry [1]. In 80% of instances [8], KIT overexpression is caused by an activating mutation in the KIT proto-oncogene. Although most GISTs are KIT positive, some KIT negative GISTs have activating mutations in PDGFRA, a similar tyrosine kinase receptor mutation [1].

Surgical resection remains the mainstay of treatment [5]. However, the use of targeted therapy of TKIs for KIT or PDGFRA mutations revolutionized the treatment of metastatic or unresectable GIST. Imatinib (STI571) was the first targeted therapy approved for the treatment of GISTs and works by attaching to the ATP-binding pocket necessary for phosphorylation and activation of the receptor; these drugs inhibit signaling via KIT or PDGFRA [9]. Imatinib is a first-line treatment for advanced GIST [1]. It is recommended to continue treatment with imatinib in patients who respond until the disease progresses or the development of intolerable side effects, as cessation of therapy can cause rapid progression of the disease [10]. Our patient was treated with imatinib, and the subsequent CT showed a reduction in masses. Tumor size, mitotic index, non-gastric location, and tumor rupture are risk factors for recurrence [4]. Immunotherapy with single-agent anti-PD1 antibodies in advance of GIST has been shown to have very limited activity [11].

The treatment recommendation for GISTs can be summarized as: resection for primary low-risk tumors; surgical resection of high-risk GISTs with adjuvant imatinib 400 mg daily; and for unresectable GISTs, neoadjuvant imatinib 400 mg daily followed by resection [10]. Sunitinib is recommended for c-Kit exon 9, 13, and 14, ponatinib is recommended for c-Kit exon 17 mutations, and regorafenib is recommended for treatment-resistant cancers [10]. Avaprinitib is recommended over imatinib for patients with PDGFRA D842V mutations [12,13]. Larotrectinib and entrectinib are recommended for GIST with the neurotrophin receptor tyrosine kinase (NTRK) mutation, which is present in a minority of GIST without KIT or PDGFRA mutations [14,15].

## Conclusions

Peritoneal carcinomatosis is a rare presentation of GIST. GIST should be considered in the differential diagnosis of intraabdominal tumors. It is important to keep in mind that the diagnosis of GIST can be challenging as the clinical presentation and imaging findings are not distinct. Surgical resection remains first-line therapy, while TKIs offer survival benefits for patients with locally advanced or metastatic GIST according to their mutation status.
